# Measurement of beta-amyloid peptides in specific cells using a photo thin-film transistor

**DOI:** 10.1186/1556-276X-7-72

**Published:** 2012-01-06

**Authors:** Chang-Beom Kim, Cheol-Joo Chae, Hye-Rim Shin, Ki-Bong Song

**Affiliations:** 1 IT Convergence Services Core Research Team, Electronics and Telecommunications Research Institute, 218 Gajeong-ro, Yuseong-gu, Daejeon, 305-700, South Korea

**Keywords:** Alzheimer's disease, beta-amyloid, photosensitive field-effect transistor, arsenic trisulfide optical filter

## Abstract

The existence of beta-amyloid [Aβ] peptides in the brain has been regarded as the most archetypal biomarker of Alzheimer's disease [AD]. Recently, an early clinical diagnosis has been considered a great importance in identifying people who are at high risk of AD. However, no microscale electronic sensing devices for the detection of Aβ peptides have been developed yet. In this study, we propose an effective method to evaluate a small quantity of Aβ peptides labeled with fluorescein isothiocyanate [FITC] using a photosensitive field-effect transistor [p-FET] with an on-chip single-layer optical filter. To accurately evaluate the quantity of Aβ peptides within the cells cultured on the p-FET device, we measured the photocurrents which resulted from the FITC-conjugated Aβ peptides expressed from the cells and measured the number of photons of the fluorochrome in the cells using a photomultiplier tube. Thus, we evaluated the correlation between the generated photocurrents and the number of emitted photons. We also evaluated the correlation between the number of emitted photons and the amount of FITC by measuring the FITC volume using AFM. Finally, we estimated the quantity of Aβ peptides of the cells placed on the p-FET sensing area on the basis of the binding ratio between FITC molecules and Aβ peptides.

## Introduction

Since the discovery of Alzheimer's disease [AD] in 1906, numerous AD researches have grown intensively from many angles during the past several decades [1-4]. Recently, the importance of early clinical diagnosis has been recognized to diagnose people at high risk of AD. According to the established hypotheses on AD during the past decade, the extracellular deposits of beta-amyloid [Aβ] peptides forming plaques and the intracellular neurofibrillary tangles have been regarded as the major histopathological hallmarks of AD [5-10]. Biologically, more evolved studies examined that Aβ peptides which are incorporated into planar lipid bilayers of neurons induce multimeric ion channels inducing excessive calcium influx into neurons and subsequent neuritic degeneration and death [11]. Another hypothesis suggested that soluble Aβ oligomers are the origin of neurotoxicity before Aβ aggregation process proceeds further and forms plaques [12].

As an Aβ detection tool, highly sophisticated neuroimaging techniques have been developed such as single photon emission computed tomography [13] and positron-emission tomography [14]. However, the neuroimaging systems have rather limitations in spatial resolution for the identification of nanoscale Aβ with a molecular-level precision because their detection depends on the computed images of Aβ plaque clumps. Recently, with rapid development of microtechnology, the incorporation of microfabricated devices with biochemical analysis techniques has been dramatically increased. Antibody-conjugated microbead arrays on a substrate were used for a multiplexed detection of several types of proteins in a microelectrophoretic device, which was, however, also on the basis of a fluorescence imaging technique [15]. Surface-enhanced Raman scattering spectroscopy was utilized to detect biomolecules in a label-free way when electrokinetically preconcentrated to amplify the low concentrations, which is also difficult for accurate quantification.

As a result of the recent AD researches, the *in vivo *physiological quantity of Aβ peptides has been known to be a few nanomoles in concentration. Therefore, the development of biosensors enabling the accurate quantification of a small amount of Aβ peptides ranging from a few femtomoles to nanomoles is required. These challenging biosensors with the capability of Aβ peptide quantification at a low concentration have not been developed yet for early AD diagnosis. Thus, this study suggests a new approach capable of evaluating a small quantity of Aβ peptide using a simple, thin-film field-effect transistor and shows the results of the photocurrents resulted from a fluorescence signal.

## Methods

### Photosensitive field-effect transistor

**Figure 1:**
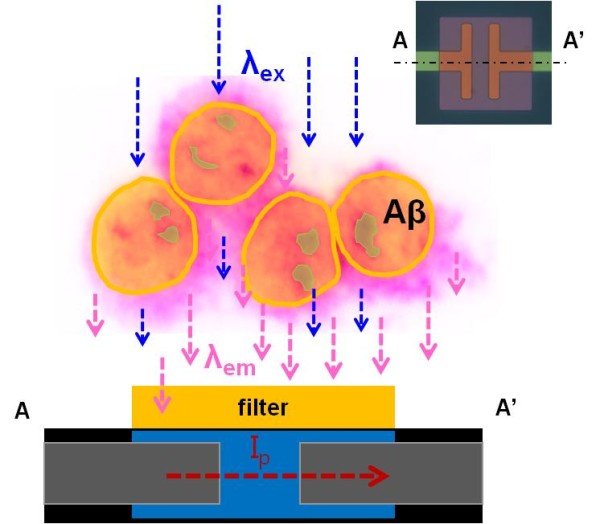
**Conceptual illustration of a p-FET**. The p-FET is integrated with an on-chip optical filter composed of a selectively transmissible material at a particular wavelength to evaluate a small quantity of Aβ peptides conjugated with FITC. The unwanted range of excitation light (*λ*_ex_) is reflected, and only the fluorescent light emitted at a proper wavelength (*λ*_em_) is transmitted through the filter and converted to the electrical signal. The inset shows the actual p-FET device.

As shown in Figure 1, we propose an effective method to measure the quantity of Aβ peptides labeled with fluorescein isothiocyanate [FITC] using a photosensitive field-effect transistor [p-FET] with an on-chip single-layer optical filter. The *in vitro *microsystem mainly consists of an upper biofluidic part where the cultured cells are introduced on top of the optical filter through a polydimethylsiloxane [PDMS] channel and an underlying thin film p-FET as the signal transducer part which was fabricated with an optically high-efficient amorphous Si [α-Si] layer on a Si/SiO_2 _substrate. A droplet including cells with intra/extracellular Aβ peptides is placed on the laminin-coated sensing area of the p-FET device, which is, after overnight incubation at 37°C, followed by the treatment of both the primary antibody for Aβ peptides and secondary antibody conjugated with fluorochrome.

### Mathematical approach

In Figure 1, the fluorochrome molecules bound to the Aβ peptides are excited by blue laser irradiation (λ_ex_) at a high energy level and emit visible fluorescent light (λ_em_) at a low energy level. Through this process, the filter surface unoccupied by the cells reflects the unwanted range of blue laser irradiation and only transmits the fluorescently emitted light through the filter. When the fluorescent light (dotted red line) emitted by the excitation (dotted blue line) is transmitted through the optical filter and approaches the p-FET sensing area (i.e., α-Si surface), excess charge carriers are generated by the absorbed photons within the α-Si sensing layer to give rise to a measurable photocurrent, which can be theoretically expressed as follows:

(1) $$I_{\text{p}}=\left(\frac{e}{hv}\right)\left(1-R\right)\eta \left(1-e^{-\alpha vd}\right)P_{\text{FET}}=\left(\frac{e}{hv}\right)C_{\text{FET}}P_{\text{FET}},$$

where *I*_p _is the photocurrent generated by p-FET, *e *is the electron charge, *h *is the Planck constant, and *ν *is the photon frequency of fluorescence emission. The remaining parts of Equation 1 represent the optical properties related to the p-FET device which can be designated as a single constant, *C*_FET_, *R *is the reflectance, *η *is the quantum efficiency, *α *is the absorption coefficient of the photon, and *d *is the p-FET sensing layer thickness. Therefore, *I*_p _is directly proportionate to *P*_FET _which represents the intensity of the incident fluorescent light transmitted through the filter. Also, the intensity of the fluorescent transmittance onto the p-FET is linearly proportional to the multiplication of the intensity of emitted fluorescence (*P*_em_) from fluorochrome before transmission and the transmittance coefficient (*T*) of the optical filter for the emitted wavelength:

(2) $$P_{\text{FET}}=T\;P_{\text{em}}.$$

Meanwhile, *P*_em _definitely relies on the number of the emitted photons from the fluorochrome excited by the blue laser. By the definition of the quantum yield as follows:

(3) $$\Phi \equiv \frac{\text{\# of emitted photons}}{\text{\# of absorbed photons}},$$

the number of emitted photons indicates the multiplication of the quantum yield and the number of photons absorbed within the fluorochrome. Subsequently, *P*_em _can be mathematically expressed as follows:

(4) $$P_{\text{em}}=\Phi \times \left(\text{\# of absorbed photons}\right)=\Phi \left(1-e^{-A_{\lambda }}\right),$$

where *A*_λ _is the absorbance of fluorochrome for the specified wavelength which is defined as the logarithmic value of the ratio of the intensity of light passed through fluorochrome to the intensity of incident light. Therefore, Equation 1 can be completely rewritten as follows:

(5) $$I_{\text{p}}=\left(\frac{e}{h\nu }\right)C_{\text{FET}}T\Phi \left(1-e^{-A_{\lambda }}\right)=C\left(1-e^{-A_{\lambda }}\right),$$

where *C *represents an arbitrary constant. Since the absorbance, *A*_λ_, is proportionate to the volume (or mass, or thickness) and the concentration of the absorbing species, Equation 5 meaningfully indicates that high electrical current may be generated by the p-FET device for a large amount of the Aβ-conjugated fluorochrome and vice versa. Eventually, since the quantity of Aβ peptides is directly proportionate to that of the tagged fluorochrome, the photocurrents generated by p-FET are a function of the amount of Aβ peptides specifically conjugated with fluorochrome.

Even though the exact relationship would not be available in a form of an equation in this study, the photocurrent generated by the p-FET device would be expressed as a linear function of the amount of the Aβ-conjugated fluorochrome and potentially provide the quantified Aβ concentration.

## Results and discussion

### Fluorochrome-conjugated Aβ expressed on a cell line

**Figure 2:**
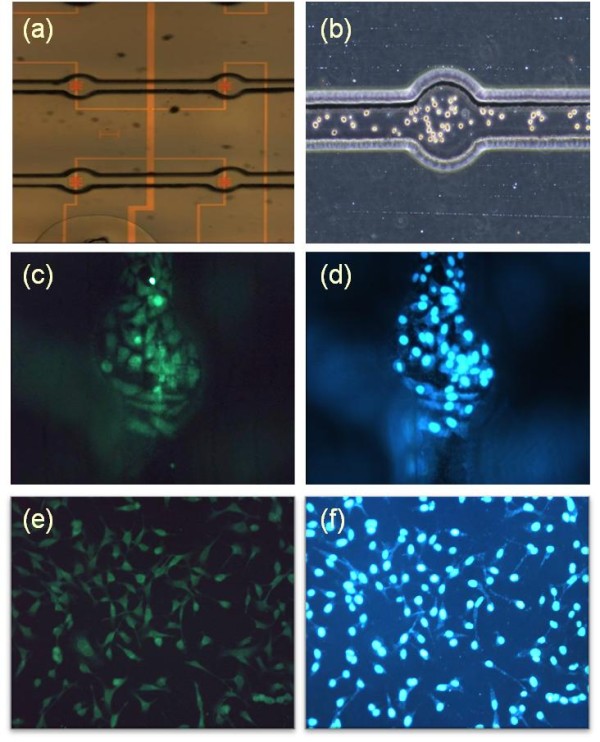
**Cell culture and visualization on the p-FET device**. (**a**) A microscopic optical image of the p-FET sensing areas placed in the middle of the PDMS well. (**b**) The HeLa cells were brought to the p-FET surface and cultured overnight in the incubator for stabilization. (**c**) The HeLa cells were visualized with FITC specifically bound to the Aβ peptides expressed on the cell surface. (**d**) The nuclei of the HeLa cells stained using DAPI. (**e**, **f**) The HeLa cells cultured on a flat PDMS surface were visualized with FITC and DAPI, respectively.

We prepared a circular PDMS well with a diameter of 200 μm to guide the biological sample solution. It was aligned on the p-FET surface, so the sensing area was placed in the middle of the well as shown in Figure 2a. After washing three times with phosphate-buffered saline, laminin as an extracellular matrix was coated on the channel, and the channel was kept overnight in an incubator at 37°C.

We prepared HeLa cells for the expression of Aβ peptides. The HeLa cells (density 10^4^/μl) were brought to the p-FET surface and cultured overnight in the incubator for stabilization as shown in Figure 2b. Thereafter, the HeLa cells were fixed with 4% paraformaldehyde for 1 h at room temperature, and then, the blocking with 4% bovine serum albumin was carried out for 1 h to protect nonspecific antibody bindings in the next step. Then, the overnight incubation with a primary antibody (Abcam, Cambridge, MA, USA) was performed for the specific binding to Aβ peptides on the HeLa cells.

The subsequent treatment with a secondary antibody (Invitrogen, Carlsbad, CA, USA) tagged with FITC was performed for 1 h (Figure 2c). The synthetic ratio of Aβ to FITC is defined to be 1:4 by the manufacturer (USBiological, Swampscott, MA, USA). After the fluorescence treatment with FITC, the nuclei of the HeLa cells were stained using 4',6-diamidino-2-phenylindole [DAPI] (Figure 2d), and the p-FET sensor was imaged using a fluorescent microscope and was irradiated by a 405-nm blue laser as an excitation source for FITC to measure the photocurrent. The reason that we used the 405-nm blue laser was attributed to the intention of forming a thin, single-layer filter on the p-FET surface, not using multilayer, commercial optical filters. We developed a thermally evaporated thin arsenic trisulfide (As_2_S_3_) filter which almost blocks the lights below 450 nm and transmits longer than approximately 500 nm. Figure 2e, f shows the typical morphologies of HeLa cells cultured on a flat PDMS surface.

### Quantification of Aβ peptides via detection of FITC

**Figure 3:**
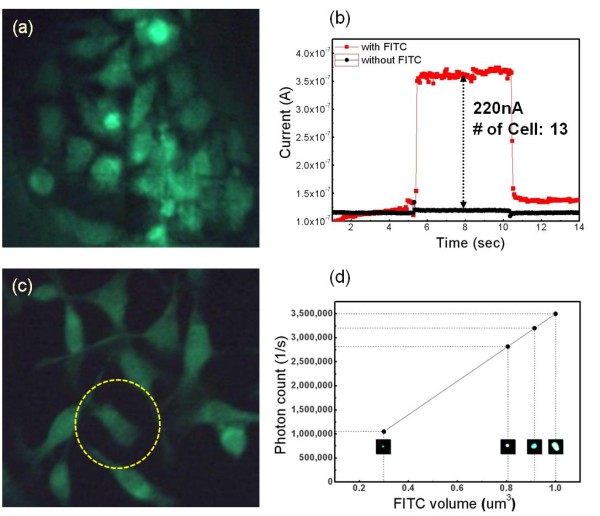
**Quantitative analysis of FITC**. (**a**) Thirteen HeLa cells placed on the p-FET sensing area in the middle of the PDMS circular well were visualized with FITC bound to the Aβ peptides expressed on the cell membranes. (**b**) The photocurrents generated by the fluorescence emitted from the FITC-conjugated Aβ peptides were measured. (**c**) The number of photons of the single cell in the yellow dotted circle was measured using a PMT. (**d**) Calibration data of the photon counting as a linear function of the FITC volume.

We investigated the applicability of our p-FET sensor to the actual detection of Aβ peptides existing in the HeLa cells. In Figure 3a, approximately 13 HeLa cells placed on the p-FET sensing area in the middle of a PDMS circular well were visualized with FITC specifically bound to the Aβ peptides expressed on the cell membranes. The peptides rather feebly fluoresced when stimulated by a blue excitation laser at a 405-nm wavelength. The photocurrents generated by the fluorescence emitted from the FITC-conjugated Aβ peptides were measured as shown in Figure 3b. Only with the optical filter layer on the p-FET (without any cells), approximately 5 nA of photocurrents (black line) were generated when the p-FET device was irradiated by the blue laser during the shutter-on (5.5 to 10.5 s), indicating that most of the excitation light was reflected by the filter layer. However, with the FITC-labeled cells on the optical filter layer, the photocurrents measured approximately 350 nA with 220-nA increments, assuming that the photocurrent generated by a single cell roughly corresponds to approximately 18 nA. Therefore, it is expected that our p-FET sensor can be used as a detector for low-level Aβ peptides with weak fluorescence emission.

Figure 3c shows the FITC-stained HeLa cells with better cellular shapes on a flat PDMS surface. To evaluate the correlation between the generated photocurrent and the number of emitted photons from a single cell, we measured the number of photons of the single cell in the yellow dotted circle using a photomultiplier tube [PMT]. As a result of the PMT measurement, a single fluorescent cell emitted approximately 1.3 × 10^6 ^photons in 1 s, which corresponds to 17 nA of photocurrent when compared to the p-FET measurement.

To accurately evaluate the quantity of Aβ peptides within a single cell as the ultimate purpose of this study, the information on how many FITC molecules exist within a single cell is imperatively required. Therefore, as shown in Equation 4, the quantum yield for FITC molecules experimentally measured 0.12, which was excited at 405 nm and emitted at 510 nm. According to both the measured quantum yield for our system and the PMT result for the photon number within a single cell (approximately 1.3 × 10^6 ^photons), the concentration of FITC molecules existing within a single cell was roughly evaluated to be approximately 10 fM. After all, the photocurrent of 220 nA measured by p-FET for 13 HeLa cells represents approximately 130 fM of FITC molecules, and the concentration of Aβ peptides is evaluated to be 40 to 50 fM on the basis of the manufacturer synthesis ratio (4:1) between FITC and Aβ. Figure 3d represents the calibration data of the photon counting using PMT measurement, showing a linear function of the FITC volume.

## Conclusions

We proposed an effective method to measure the quantity of Aβ peptides labeled with FITC using a p-FET with an on-chip single-layer optical filter. To accurately evaluate the quantity of Aβ peptides within the cells cultured on the p-FET device, we measured the photocurrents which resulted from the FITC-conjugated Aβ peptides expressed from the cells and measured the number of photons of the fluorochrome in the cells using a PMT. Thus, we evaluated the correlation between the generated photocurrents and the number of emitted photons. We also evaluated the correlation between the number of emitted photons and the amount of FITC by measuring the FITC volume using AFM. Finally, we estimated the quantity of Aβ peptides of the cells placed on the p-FET sensing area on the basis of the binding ratio between FITC molecules and Aβ peptides.

## Competing interests

The authors declare that they have no competing interests.

## Authors' contributions

C-BK and C-JC fabricated the p-FET devices, conducted the thermal coating of the optical filter, carried out the experiments, and drafted the manuscript. H-RS carried out the preparation of cells and the treatment of antibody and fluorescence. K-BS conceived the basic idea of the whole experiment and supported the mathematical approach and the organization of this article. All authors read and approved the final manuscript.
